# Relationship between Antibody Susceptibility and Lipopolysaccharide O-Antigen Characteristics of Invasive and Gastrointestinal Nontyphoidal *Salmonellae* Isolates from Kenya

**DOI:** 10.1371/journal.pntd.0003573

**Published:** 2015-03-04

**Authors:** Robert S. Onsare, Francesca Micoli, Luisa Lanzilao, Renzo Alfini, Chinyere K. Okoro, Anne W. Muigai, Gunturu Revathi, Allan Saul, Samuel Kariuki, Calman A. MacLennan, Simona Rondini

**Affiliations:** 1 Centre for Microbiology Research (CMR), Kenya Medical Research Institute (KEMRI), Nairobi, Kenya; 2 Jomo Kenyatta University of Agriculture and Technology (JKUAT), Nairobi, Kenya; 3 Novartis Vaccines Institute for Global Health (NVGH), Siena, Italy; 4 Wellcome Trust Sanger Institute, Wellcome Trust Genome Campus, Hinxton, Cambridge, United Kingdom; 5 Division of Microbiology, Department of Pathology, Aga Khan University Hospital, Nairobi, Kenya; Oxford University Clinical Research Unit, VIETNAM

## Abstract

**Background:**

Nontyphoidal *Salmonellae* (NTS) cause a large burden of invasive and gastrointestinal disease among young children in sub-Saharan Africa. No vaccine is currently available. Previous reports indicate the importance of the O-antigen of *Salmonella* lipopolysaccharide for virulence and resistance to antibody-mediated killing. We hypothesised that isolates with more O-antigen have increased resistance to antibody-mediated killing and are more likely to be invasive than gastrointestinal.

**Methodology/Principal Findings:**

We studied 192 NTS isolates (114 Typhimurium, 78 Enteritidis) from blood and stools, mostly from paediatric admissions in Kenya 2000–2011. Isolates were tested for susceptibility to antibody-mediated killing, using whole adult serum. O-antigen structural characteristics, including O-acetylation and glucosylation, were investigated. Overall, isolates were susceptible to antibody-mediated killing, but *S.* Enteritidis were less susceptible and expressed more O-antigen than Typhimurium (p<0.0001 for both comparisons). For *S.* Typhimurium, but not Enteritidis, O-antigen expression correlated with reduced sensitivity to killing (r = 0.29, 95% CI = 0.10-0.45, p = 0.002). Both serovars expressed O-antigen populations ranging 21–33 kDa average molecular weight. O-antigen from most Typhimurium were O-acetylated on rhamnose and abequose residues, while Enteritidis O-antigen had low or no O-acetylation. Both Typhimurium and Enteritidis O-antigen were approximately 20%–50% glucosylated. Amount of *S.* Typhimurium O-antigen and O-antigen glucosylation level were inversely related. There was no clear association between clinical presentation and antibody susceptibility, O-antigen level or other O-antigen features.

**Conclusion/Significance:**

Kenyan *S.* Typhimurium and Enteritidis clinical isolates are susceptible to antibody-mediated killing, with degree of susceptibility varying with level of O-antigen for *S.* Typhimurium. This supports the development of an antibody-inducing vaccine against NTS for Africa. No clear differences were found in the phenotype of isolates from blood and stool, suggesting that the same isolates can cause invasive disease and gastroenteritis. Genome studies are required to understand whether invasive and gastrointestinal isolates differ at the genotypic level.

## Author Summary

Nontyphoidal *Salmonellae* (NTS) are an emerging major cause of invasive bacterial disease in African children aged less than 5 years and immunocompromised adults, with an estimated case fatality rate of 20–25%. NTS also cause diarrhoea, a killer of about 1.5 million young children annually, mainly in low- and middle-income countries. No vaccine against NTS is available, but improved understanding of the *Salmonella* bacteria that cause disease in Africa would help the development of new vaccines. The authors characterized a collection of 192 Kenyan NTS strains (114 *S*. Typhimurium and 78 *S*. Enteritidis) from blood and stool specimens. All strains could be killed to differing extents by antibodies present in the blood of healthy HIV-uninfected African adults, supporting the development of a vaccine that will induce protective antibodies when given to African children. Differences in killing by antibody were partly related to the amount of O-antigen on the bacterial surface. There were no clear distinction between stains causing invasive disease and diarrhoea, suggesting that the same strains may be capable of causing both forms of disease. Clarification of this will require genomic analysis.

## Introduction

NTS are a major but neglected cause of invasive disease (hence iNTS disease) in Africa [1–3]. *Salmonella enterica* serovars Typhimurium and Enteritidis account for nearly 80% of all human isolates reported globally [4]. While in developed countries, these predominantly cause a mild self-limiting gastroenteritis [5–7], in Africa they are responsible for bacteraemia, often associated with meningitis in young children, with incidence rates comparable to invasive *S*. *pneumoniae* disease [3]. The true burden of iNTS disease is uncertain due to the absence of a characteristic clinical presentation. Patients often present with nonspecific fever [8–10] and blood culture is necessary for diagnosis. Even where blood culture facilities are available, rapid clinical progression of NTS bacteraemia results in many patients dying before a microbiological diagnosis can be made [10]. No vaccine is available, and clinical management is made difficult by widespread multi-drug resistance and the need for late-generation expensive antibiotics [11–13].

In Kenya, iNTS disease is particularly frequent in rural areas [14], with incidence rates as high as 568/100,000 person-years [15]. A recent study from Western Kenya found an association between NTS diarrhoea and mortality in hospitalized children [16], indicating that NTS isolates in the region can cause fatal invasive and gastrointestinal disease, but it is currently unknown whether specific microbial phenotypic or genotypic characteristics are associated with each clinical presentations. Whole genome sequencing studies demonstrate that invasive African *S*. Typhimurium are genetically distinct from those in the rest of the world, and are characterized by a new sequence type, ST313, that has spread throughout the continent [17]. The ST313 pathovar is associated with genome degradation and pseudogene accumulation [13]. Equivalent studies are ongoing for *S*. Enteritidis which is more prevalent than *S*. Typhimurium in some parts of Africa [18,19], and globally [20]. Investigation of the relationship between genotypic and phenotypic features of Typhimurium isolates is ongoing and a systematic characterization of endemic Typhimurium and Enteritidis isolates from one endemic region is awaited.

Lipopolysaccharide (LPS) forms the outer layer of *Salmonella* and other Gram-negative bacteria, and is key to the interaction between *Salmonella* and its environment. The O-antigen chain (including core sugars, hereafter referred to as ‘OAg’) constitutes the outermost part of LPS [21]. In pathogenic bacteria such as *Salmonella*, LPS plays an important role in the interaction between the bacterium and its host and is a virulence factor required for colonization and resistance to antibody-mediated killing [22]. While the lipid A moiety is the predominant cause of the endotoxic effects of LPS, OAg is the most immunodominant portion of the molecule, and is responsible for serovar-specificity [23]. OAg protects bacteria from the environment and from serum complement [24–26], which can lead to bacterial killing by membrane attack complex formation. Rough strains of *Salmonella* that lack OAg are avirulent and succumb readily to complement-mediated killing [26]. The OAg structure plays a role in bacterial virulence, with longer OAg chains associated with increased complement and antibody resistance [27–29] and protection against other host antimicrobial factors [30].

In this study we analysed a bacterial collection of 114 *S*. Typhimurium and 78 *S*. Enteritidis isolates derived from patients, mostly children, presenting to three main Kenyan hospitals in Nairobi and from 31 healthy carriers related to these patients. Our primary objective was to characterise NTS isolates circulating in endemic areas, with the goal of identifying bacterial features associated with either invasive or gastrointestinal pathology. We hypothesised that invasive isolates would, by nature, be more resistant to survival in the blood than gastrointestinal isolates, and focused our analysis on the susceptibility of isolates to antibody-mediated killing and characterization of the OAg expressed by the various isolates. We investigated the total amount of OAg expressed by the various isolates and OAg specific characteristics such as molecular weight (MW), O-acetylation and glucosylation levels, parameters which can influence OAg immunogenicity. Additionally, we tested the sensitivity of the isolates to antibody killing, using whole human serum from HIV-uninfected adults [31]. The results obtained were compared with clinical presentation in order to identify possible associations.

## Materials and Methods

### Origin of bacterial isolates and serum samples

The study culture collection contained 192 archived NTS isolates (114 *S*. Typhimurium and 78 *S*. Enteritidis) collected between 2000 and 2011 at the Centre for Microbiology Research (CMR), Kenya Medical Research Institute (KEMRI) in Nairobi, Kenya. Study isolates were selected from the archived bacterial culture collection based on availability of unequivocal serovar identification and availability of clinical records and metadata. The isolates were stored in Tryptic Soy Broth with 15% glycerol at-80°C. The isolates were from blood and stools with a few additional samples from urine (3 samples), cerebrospinal fluid (CSF, 2 samples) and environmental sources (2 samples). The isolates were mostly from children admitted to three main hospitals in Nairobi county, namely: Kenyatta National Referral hospital, Gertrude’s Children’s Hospital and Aga Khan University Hospital. This collection also included isolates from healthy carriers (31 samples). Before use in the present study, the archived isolates were re-cultured and the serotype was confirmed by antibody-based agglutination (Bio-Rad Laboratories, Inc., Hercules, CA, USA).

Based on the clinical sources and sample type, the NTS isolates were divided into four main groups. Those isolates from body fluids that are normally sterile such as blood and CSF were grouped as ‘invasive’, those from stool samples of patients were grouped as ‘gastroenteric’, those from stool samples of healthy individuals were grouped as ‘healthy carriers’ while those from soil and sewer water sources were grouped as ‘environmental’. The three isolates from urine samples were considered clinically significant invasive sterile site isolates since *Salmonellae* are not part of the normal perineal skin flora and the isolates were from patients with symptoms of urinary tract infection.

Undiluted sera from ten healthy HIV-uninfected Malawian adults were used to generate a pooled serum to assess sensitivity to antibody-mediated killing of the *Salmonella* isolates. Each serum was tested prior to pooling to ensure that killing of the index *S*. Typhimurium ST313 strain, D23580, was within the 0.9 to 3.0 Log_10_ range previously described for such sera [32]. Sera were handled at 4°C during the pooling process to ensure preservation of endogenous complement activity and frozen in aliquots at-80°C. Ethical approval for preparation of this serum was granted by the College of Medical Research and Ethics Committee, College of Medicine, University of Malawi [31].

### Serum Bactericidal Activity (SBA) assay

The study isolates were tested for their susceptibility/resistance to killing against the serum pool described above, using a protocol involving whole serum and endogenous complement activity as previously described [26]. Briefly, 5 μl washed bacteria at 2 h log-growth phase at an OD of approximately 0.2 (with shaking at 180 rpm) was added to 45 μl undiluted serum at a final bacterial concentration of 1×10^6^ colony forming units, CFU/ml and incubated at 37°C with the number of viable bacteria count determined by serial dilution on Luria Bertani (LB) agar after 0 and 180 min. As a negative control, reference sera were heat-inactivated at 56°C for 45 min to inactivate endogenous complement and included in each SBA experiment. Additional internal controls included testing the representative endemic African *S*. Typhimurium isolate D23580, previously shown to be susceptible to serum killing [26] and hyperimmune mouse serum containing high levels of anti-OAg antibodies [33]. Susceptibility to serum killing was determined as any reduction in viable bacterial count compared with the initial *Salmonella* concentration.

### O-antigen extraction and quantification

OAg extraction was performed by acid hydrolysis [34]. Bacterial isolates were grown overnight in LB medium. As OAg expression can be influenced by growth conditions, identical conditions were used for the growth of all strains [29]. The bacterial OD was measured and the bacterial cultures were concentrated in PBS to OD: 35. Acetic acid (2% v/v) was then added to the concentrated growth bacterial culture (pH 3), which were incubated for 3h at 100°C. The reaction was stopped by the addition of 14% ammonium hydroxide, increasing the pH to around 6. Bacterial debris was removed by centrifugation and OAg in the supernatant was 0.22 μm microfiltered and desalted by HiTrap Desalting 5 ml columns (GE Healthcare, UK). Phenol sulphuric acid assay, using glucose as standard, was used for quantification of OAg content [34,35].

### ^1^H Nuclear Magnetic Resonance Spectroscopy (^1^H NMR)

^1^H NMR analysis was performed as a confirmation of the identity of the OAg samples and to verify the presence of O-acetyl groups along the OAg chain as previously described [34].

### High Performance Anion Exchange Chromatography with Pulsed Amperometric Detection (HPAEC-PAD)

Glucosylation level of OAg samples was estimated by HPAEC—PAD after acid hydrolysis of the OAg to release the monosaccharides constituting the sugar chain as described previously. Commercial glucose was used for building the calibration curve (0.5–10 μg/ml). Glucose (Glc) level is calculated as molar ratio of Glc to rhamnose (Rha), sugar present in each OAg repeating unit. The analysis was also used as confirmation of the OAg samples identity verifying the correct monosaccharides ratios in the repeating unit [34,36].

### Size-exclusion High-Performance Liquid Chromatography (HPLC-SEC)

HPLC—SEC analysis was used to estimate the molecular size distribution of OAg populations [34,36]. Samples were run, without pre-treatment, on a TSK gel G3000 PWXL column (30 cm x_7.8 mm; particle size 7 um; cod. 808021) with a TSK gel PWXL guard column (4.0 cm _x 6.0 mm; particle size 12 um; cod. 808033) (Tosoh Bioscience, Tokyo, Japan). The mobile phase was 0.1 M NaCl, 0.1 M NaH_2_PO_4_, 5% CH_3_CN, pH 7.2, at the flow rate of 0.5 ml/min (isocratic method for 30 min). OAg peaks were detected by differential refractive index (dRI). Void and bed volume calibration was performed with λ-DNA (λ-DNA molecular weight (MW) Marker III 0.12–21.2 kb; Roche) and sodium azide (Merck, New Jersey, USA), respectively. OAg average MW was estimated on standard dextrans (Sigma) calibration curve.

### Statistical analyses

Serum susceptibility and OAg amounts of the two *Salmonella* serovars examined (*S*. Typhimurium and *S*. Enteritidis) was compared using the Mann-Whitney U test. Possible correlations (i.e. OAg amount and serum susceptibility, glucosylation levels and OAg production) were analysed by Spearman rank.

## Results

### NTS bacterial collection

Of the 192 NTS isolates in the study, 114 (59%) were *S*. Typhimurium and 78 (41%) were *S*. Enteritidis. This study collection included isolates from blood, stools (patients and healthy contacts), CSF, urine and environmental/animal sources as described in Table 1 (see S1a-b Table for supporting information). The isolates were obtained from hospitals based in Nairobi, an urban setting. Clinical information on patients’ age was available for 67% of cases (73 with *S*. Typhimurium, 57 with *S*. Enteritidis). The age ranged from 1 month to 59 years for *S*. Typhimurium isolates and 0 months to 64 years for *S*. Enteritidis isolates, the majority (65%, 85 isolates) of these patients being below 5 years (53 *S*. Typhimurium, 32 *S*. Enteritidis). Overall, the median age of subjects from whom *S*. Typhimurium and *S*. Enteritidis strains were isolated was 2 and 3.5 years, respectively. Ages were not available for the healthy carriers.

**Table 1:** Number of study NTS isolates by serovar and origin.

	*S*. Typhimurium (% of total *S*. Typhimurium, n = 114)								*S*. Enteritidis (% of total *S*. Enteritidis, n = 78)				Total (% of total NTS)
	blood	Stool	blood & stool	urine	CSF	Soil	Sewer water	ND	blood	Stool	blood & stool	ND	
Invasive	48 (42%)	−	7 (6%)	3 (3%)	2 (2%)	−	−		22 (28%)	−	5 (6%)	−	87 (45%)
Gastrointestinal	−	24 (21%)	−	−	−	−	−	−	−	32 (41%)	−	−	56 (29%)
Healthy carriers	−	21 (18%)	−	−	−	−	−	−	−	10 (13%)	−	−	31 (16%)
Environmental	−	−	−	−	−	1 (1%)	1 (1%)	−	−	−	−	−	2 (1%)
ND	−	−	−	−	−	−	−	7 (6%)	−	−	−	9 (12%)	16 (8%)
**Total**	**114**								**78**				**192**

ND = Isolates for which no documentation on clinical form was available; n = Total number

Gastrointestinal and healthy carrier isolates were derived from stool samples.

### SBA results

**Fig 1:**
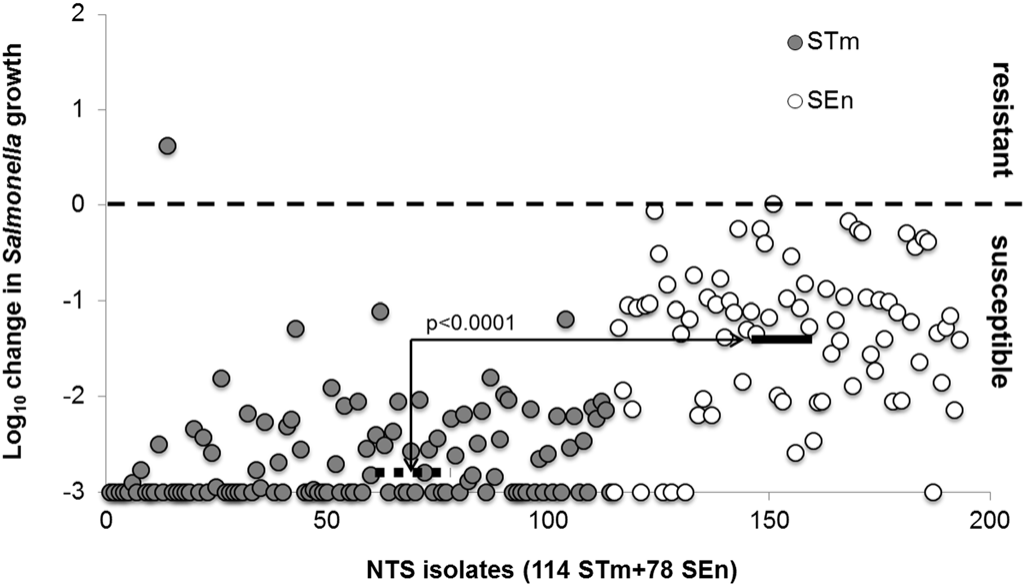
SBA results of STm and SEn isolates with Malawi serum pool. Bacterial colony forming units (CFU) were counted at time 0 (T0) and after 3 h (T180) incubation of bacteria in 45 μL undiluted serum (final bacterial concentration 1×10^6^ CFU/ml). Killing/growth was determined by Log_10_ (CFU at T180)-Log_10_ (CFU at T0). Strains that could grow (Log_10_ change >0) were considered “resistant”, strains that were killed (Log_10_ change <0) were considered susceptible. Dashed/continuous line indicates median Log_10_ change of STm (-2.9) and SEn (-1.2) isolates, respectively.

SBA results using the serum pool demonstrated a marked difference in susceptibility to antibody-mediated killing among individual isolates and a clear difference in susceptibility between *S*. Typhimurium and *S*. Enteritidis isolates (Fig. 1, and see S2a and S2b Table for supporting information).). We have previously demonstrated that this killing is through the antibody-dependent complement-mediated mechanism and requires the presence of specific antibodies and intact complement function [26]. We have also demonstrated that the control sera used in this assay from healthy Malawian adults contains abundant antibody levels to NTS [32]. All except one *S*. Typhimurium isolate (0.9%, 1/114) underwent a reduction in viable bacterial counts over the 180 minute time course of the assay, and so were designated ‘sensitive’ to antibody-mediated killing. Overall median Log_10_ reduction in viable bacteria after 180 minutes was 2.9 for *S*. Typhimurium. Indeed 50% (57/114) *S*. Typhimurium were highly susceptible to killing, undergoing a 3.0 Log_10_ reduction. In contrast *S*. Enteritidis as a group were less susceptible to antibody-mediated killing than *S*. Typhimurium, being killed by a median of 1.2 Log_10_ (p<0.0001, Mann Whitney test). However, all *S*. Enteritidis were sensitive to killing. A small proportion (10.3%, 6/78) was killed by a full 3.0 Log_10_.

### OAg characterization

**Fig 2:**
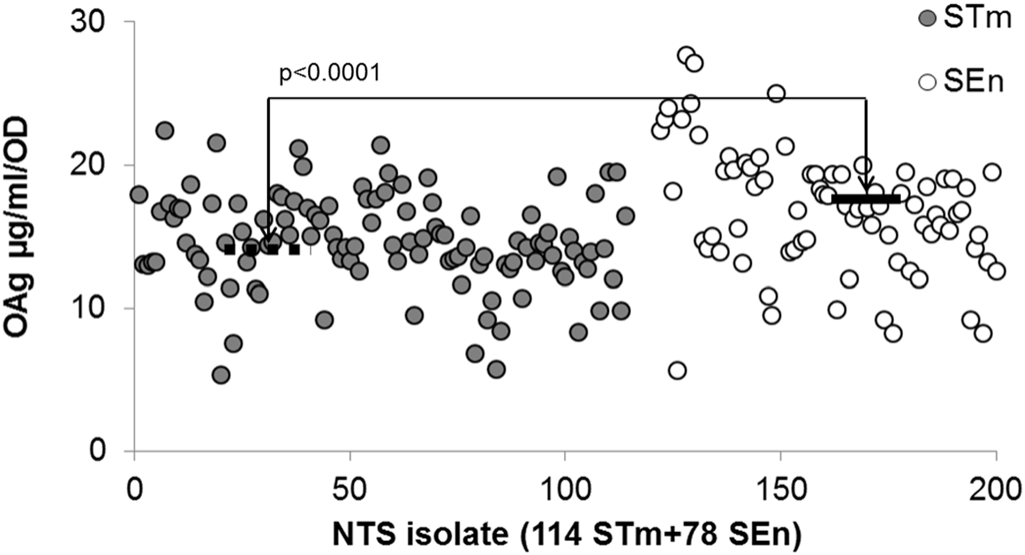
OAg production of *S*. Typhimurium (STm) and *S*. Enteritidis (SEn) isolates after growth and normalization to the same final OD: 35. Dashed/continuous line indicates median OAg production of *S*. Typhimurium (STm) (14.4 μg/ml/OD) and *S*. Enteritidis (SEn) (16.8 μg/ml/OD) isolates, respectively.

**Fig 3:**
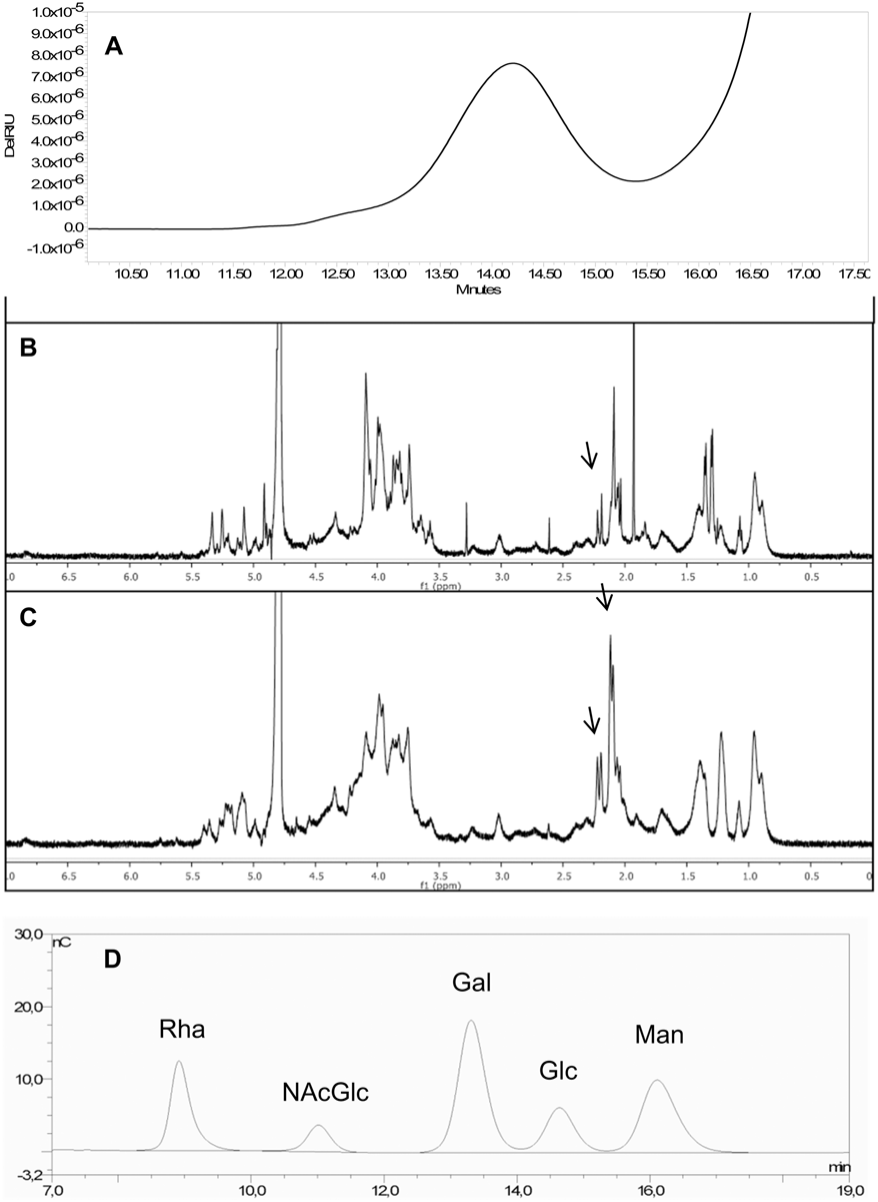
a) HPLC-SEC profile of *S*. Typhimurium Ke212 OAg (similar profiles obtained for all NTS strains; b) ^1^H NMR of *S*. Enteritidis Ke189 OAg and c) *S*. Typhimurium Ke004 OAg (arrows indicate O-acetyl groups); d) HPAEC-PAD profile of *S*. Typhimurium Ke012 OAg sample indicating typical sugars of the repeating unit (Rha, Gal, Glc and Man) and of the core region (Gal, Glc and NAcGlc) (similar profiles obtained for all NTS strains).

OAg was extracted from all 192 NTS study isolates normalized to the same final OD and total amount was quantified by phenol sulphuric acid assay [34]. NTS isolates produced a range of OAg amounts, with low (<10 μg/ml/OD) and high producers (>25 μg/ml/OD). On average, *S*. Enteritidis isolates expressed more OAg than *S*. Typhimurium, with 70.9% of the isolates producing more than 15 μg/ml/OD OAg compared to only 42.1% *S*. Typhimurium. Median OAg production for *S*. Enteritidis isolates was 16.8 μg/ml/OD compared to 14.4 μg/ml/OD of *S*. Typhimurium (p<0.0001 by Mann Whitney test) (Fig. 2). All study isolates contained one single main OAg population with average MW of 21–33 kDa (Fig. 3a); <5% isolates contained OAg population with average MW < 6 kDa.

NMR analysis on a set of 118 samples (73 *S*. Typhimurium and 45 *S*. Enteritidis) showed that only 6.8% of *S*. Typhimurium OAg were not O-acetylated, compared with 35.6% and 42.2% of *S*. Enteritidis OAg with no or traces of O-acetyl groups, respectively. For *S*. Typhimurium, OAg, O-acetylation was detected on both rhamnose (Rha) and abequose (Abe) sugar residues in the majority of isolates (63%), but also on Rha only (9.6%) and Abe only (17.8%) (Table 2; Fig. 3b and c; see S3a Table for supporting information). HPAEC-PAD was done on a subset of 91 isolates (55 S. Typhimurium and 36 SEn) and showed that for both serovars, the majority of strains (72.2%) expressed OAg with 20–50% glucosylation levels (Fig. 3d, see S3b Table for supporting information).

**Table 2:** Proportion distribution in percentages of O-acetylation and glucosylation levels among NTS isolates.

	O-Acetylation (n = 73 *S*. Typhimurium, 44 *S*. Enteritidis)					Glucosylation (n = 55 *S*. Typhimurium, 36 *S*. Enteritidis)		
	% strains with O-acetyl groups			% strains with traces or no O-acetyl groups		% strains with glucosylation level		
	On both Rha & Abe	On Abe only	On Rha only	With traces of O-acetyl groups	No O-acetyl groups	<20%	20–50%	>50%
***S*. Typhimurium**	63.0	17.8	9.6	3.0	6.8	29.1	56.4	14.5
***S*. Enteritidis**	22.2			42.2	35.6	13.9	72.2	13.9

n = Total number; Rha = rhamnose; Abe = abequose

### Analysis of associations between clinical presentation and bacterial characteristics

**Fig 4:**
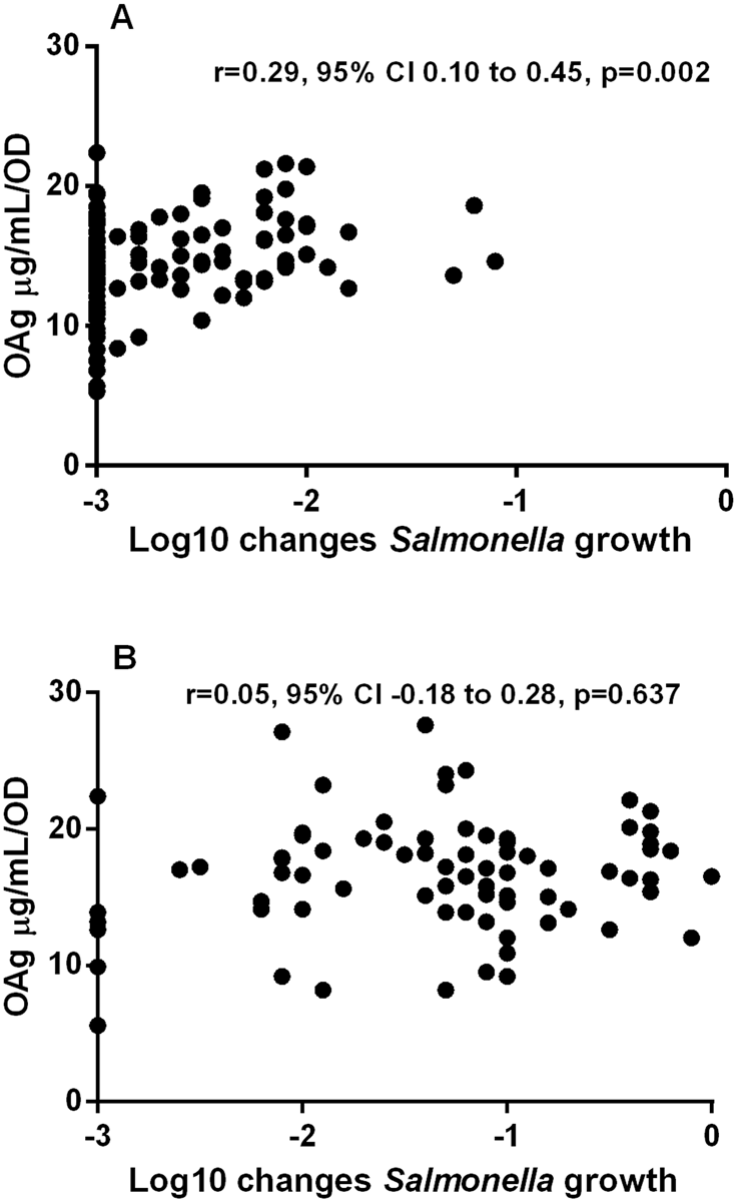
Correlation of SBA results and OAg production levels in (a) *S*. Typhimurium isolates (p = 0.002) and (b) *S*. Enteritidis isolates (p = 0.637). r is the Spearman correlation coefficient. CI is confidence interval.

SBA results, OAg features and clinical presentation were compared. As mentioned above, there was a clear inter-serovar difference, with *S*. Enteritidis isolates having more OAg than *S*. Typhimurium and being more resistant to antibody-mediated killing than *S*. Typhimurium. Among *S*. Typhimurium isolates, there was a correlation between amount of OAg and resistance to antibody-mediated killing (Spearman r = 0.29, 95% CI 0.10 to 0.45, p = 0.002) in accordance with our initial hypothesis, but this was not present for *S*. Enteritidis (Fig. 4).

**Fig 5:**
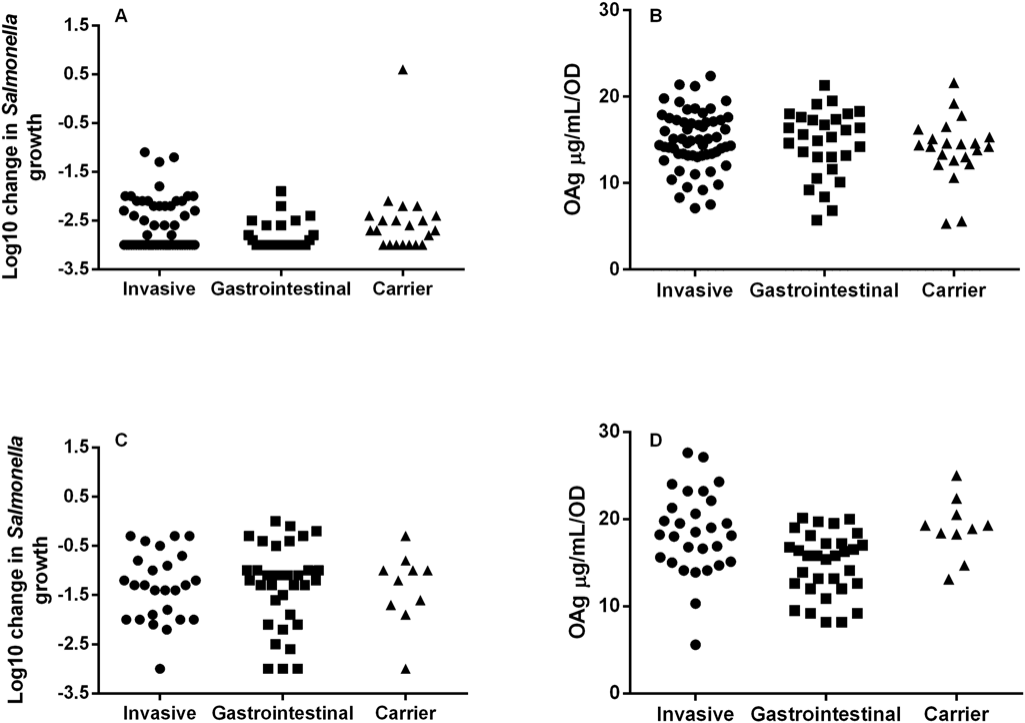
Correlation of SBA results (a, c) and OAg production levels (b, d) in *S*. Typhimurium (a, b) and *S*. Enteritidis (c, d) isolates with clinical presentations. Samples for which no clinical presentation was determined were excluded from analysis. Malawi serum pool was used for SBA. Invasive: *Salmonella* isolates from blood, urine, CSF. Gastrointestinal: *Salmonella* isolates from stools. Controls: *Salmonella* isolates from stools of healthy controls.

**Fig 6:**
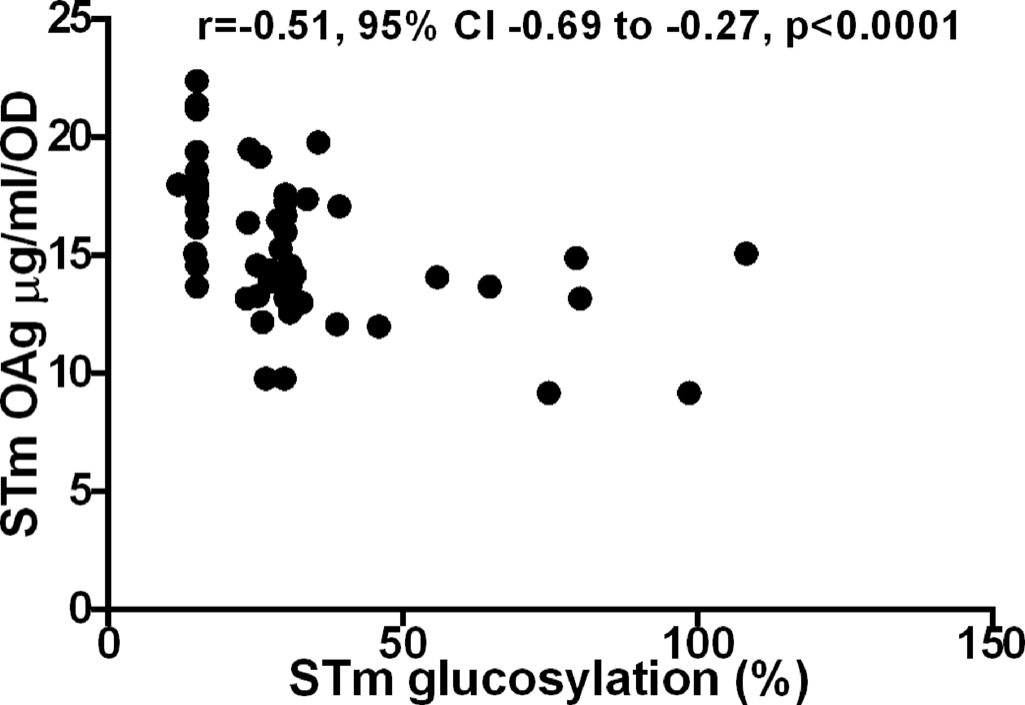
Correlation of *S*. Typhimurium (STm) OAg production levels and glucosylation (p<0.0001). r is the Spearman correlation coefficient. CI is confidence interval.

No correlation was found between NTS clinical presentation (invasive, gastrointestinal and carrier) and either antibody susceptibility or polysaccharide production, for either *S*. Typhimurium or *S*. Enteritidis isolates (Fig. 5). There was no clear correlation between O-acetylation levels/position and glucosylation levels with clinical presentation, antibody susceptibility and OAg production. For *S*. Typhimurium, we found an association between lower glucosylation levels and higher OAg production (Spearman r = 0.51, 95% CI-0.69 to-0.27, p value<0.001) (Fig. 6).

## Discussion

NTS infections are a major problem in sub-Saharan Africa and can either present as bacteraemia, with symptoms of gastroenteritis in under half of patients, or as diarrheal disease without bloodstream infection [1,11,37]. Both presentations can be life-threatening, with case fatality rates of 20–25% for iNTS bacteremia in children [1,2,8,10] and up to 50% in HIV-infected adults [9], and a recognized association between NTS diarrhoea with mortality [16]. No vaccine is currently available.

The main finding of the study is that all isolates studied, apart from one *S*. Typhimurium, were susceptible to antibody-dependent complement-mediated killing, using a serum pool from an endemic region. Previously it has been shown that the antibody response specific to NTS is acquired with age, corresponding with a decline in cases of NTS bacteraemia [26,38]. The serum pool we used contained high levels of anti-OAg (both *S*. Typhimurium and *S*. Enteritidis) antibodies as expected among healthy adults in a *Salmonella* endemic region of sub-Saharan Africa such as Kenya, Malawi or Tanzania [32]. The study therefore confirms the bactericidal activity of anti-*Salmonella* antibodies against the vast majority of endemic NTS isolates in the current study. These findings support an important role for antibodies in protective vaccines against iNTS disease [31,33,39].

Another key finding is the difference in relationship between susceptibility to antibody-mediated killing and levels of OAg expression for the two *Salmonella* serovars studied. According to our study hypothesis, decreased susceptibility of *S*. Enteritidis compared with *S*. Typhimurium could be the result of higher levels of OAg, not excluding the contributions of other pathogen features, not investigated here. A previous report from Malaysia found that *S*. Enteritidis isolates were more often isolated from the blood than stool of patients compared with other *Salmonella* serovars [40], but the reasons for this were not explored. Considering that OAg length and density play a role in pathogen virulence [27], increased levels of OAg expression may enhance the ability of *S*. Enteritidis isolates to evade host first line defence mechanisms and gain access to the bloodstream. It has also been reported that, when exposed to serum, *S*. Typhimurium upregulates genes for increased production of very long chain OAg to escape complement-dependent killing [28]. Our finding of a correlation between OAg expression and resistance to antibody-mediated killing among *S*. Typhimurium isolates is consistent with the hypothesis that high OAg expression protects against such killing. It is also well know that strains lacking OAg are rapidly eliminated from the host [41,42], and there were no such isolates in our collection. However, the lack of correlation between OAg expressed by *S*. Enteritidis isolates and susceptibility to antibody-mediated killing does not support our hypothesis for this serovar concerning OAg levels and resistance to antibody killing. This finding suggests that other inherent factors of *Salmonella*, particularly *S*. Enteritidis, have a role in determining loss of sensitivity. In other words, quantity of OAg is more important for resistance to antibody-killing of *S*. Typhimurium than *S*. Enteritidis. Further studies are required to better understand the factors contributing to the inherent resistance of *S*. Enteritidis.

In relation to OAg fine specificity (O-acetylation) of the isolates in our collection, *S*. Typhimurium were more heterogeneous than *S*. Enteritidis. OAg O-acetylation occurred in almost all *S*. Typhimurium isolates, and most often at two different sites: C-2 Abe (known to provide factor O:5 specificity) and C-2/C-3 Rha. Rha O-acetylation was first reported by Wollin et al [43] and we have previously reported this as part of the characterisation of an invasive Malawian *S*. Typhimurium isolate [44]. The extent to which this additional O-acetylation was widespread in our collection suggests a possible role in pathogenesis that should be investigated further. Moreover, the fact that we could also identify *S*. Typhimurium isolates with either Abe- or Rha-O-acetylation only, shows that O-acetylation at both sites can occur independently. A *S*. Typhimurium OAg-based vaccine for Africa would need to induce antibodies capable of binding to both OAg specificities. In contrast, O-acetylation of *S*. Enteritidis OAg was not as common, with more than two-thirds of the isolates lacking or having very low levels of O-acetylation.

Glucosylation is another OAg modification for which variation has been described for *S*. Typhimurium and can occur on the O:12 antigen galactose (C4 position) generating the 12–2 variant, and on the O:1 antigen galactose (C6 position) (phage-determined) [45]. It has been reported that O:12 glucosylation in Typhimurium is not constitutive, occurs during intracellular macrophage growth and is associated with enhanced intestinal colonization [46]. All *S*. Typhimurium isolates in our collection were glucosylated, with less than 30% of them at levels < 20%, showing that glucosylation is a very common feature among endemic strains. Similarly, 86% *S*. Enteritidis isolates investigated showed a glucosylation level >20%. Glucosylation in *S*. Enteritidis is not associated with specific OAg factors and it has not been extensively studied. A report on *S*. Enteritidis food-borne salmonellosis strains showed glucosylated LPS in some mouse isolates, closely related to strains obtained from eggs [47], but not in archived *S*. Enteritidis strains.

In *Shigella*, LPS glucosylation promotes bacterial invasion by enhancing type III secretion system (T3SS) function and is associated with reduced LPS chain length [48]. In *Salmonella*, T3SS is necessary for invasion of intestinal epithelium and survival within macrophages and further investigation into different glucosylation levels and T3SS-mediated invasion is still to be performed. The inverse correlation between *S*. Typhimurium OAg glucosylation and OAg expression observed in the current study may be related to T3SS function, as previously reported for *Shigella* [48] and *S*. Typhimurium [30]. *Salmonella* isolates defective for the synthesis of long and very long OAg species have been shown to have increased translocation of a *Salmonella* pathogenicity island 1 (SPI1)-T3SS effector protein promoting invasion. Therefore, also for *Salmonella*, there may be the need to balance T3SS activity, possibly enhanced by shorter and glucosylated LPS, and protection against innate immune effectors, which is favoured by a thicker LPS layer [27,28].

The reasons for NTS disease presenting as invasive or enteric disease are not well understood and could relate to host immunity and/or pathogen virulence. Epidemiological data indicate a frequent presentation of the invasive syndrome, without diarrhoea symptoms, in younger age groups [49], often associated with other infections such as malaria and HIV [50–52]. However, specific investigations on the relationship between invasive and diarrheal disease are extremely limited, partially due to the limited microbiological resources in endemic African countries and few data are available on contemporaneous presence of NTS in stool and blood samples [53]. Another problem is that the concentration of *Salmonella* in the blood of bacteraemic patients can be as low as 1 CFU/ml [54] and blood culture sensitivity is estimated to be about 50% [54].

The lack of obvious difference between phenotype of NTS isolates from invasive and gastrointestinal disease episodes is consistent with the concept that invasive NTS isolates are able to cause diarrhoea and vice versa. If true, this would implicate host immunity as the major determinant of NTS disease presentation in endemic sub-Saharan African countries. Other previous studies [49] have not managed to identify specific pathogen determinants for either invasive or gastrointestinal syndromes, therefore the interplay between host and pathogen may be key.

There are several limitations to the current study: categorization of isolates as “invasive” or “gastroenteric” was based on clinical presentation, as confirmed by microbiological analysis. Blood and stool samples belonging to the same patient were rarely collected, making it impossible to exclude that bacteria from bacteremic patients were not also present in stool and vice versa [53]. Another limitation was the lack of patients’ outcome data, which could give further insights into possible differences in serovar- and isolate pathogenicity,

In summary, Kenyan NTS isolates from patients with bacteraemia and gastroenteritis were susceptible to antibody-mediated killing, supporting the development of an antibody-inducing vaccine against NTS for Africa. *S*. Enteritidis were generally less susceptible to killing than *S*. Typhimurium isolates and expressed higher levels of OAg, but while OAg expression correlated with antibody resistance for *S*. Typhimurium isolates, no correlation could be found for *S*. Enteritidis, supporting a role for other inherent bacterial factors in conferring resistance to antibody killing for this *Salmonella* serogroup. Serovar- and strain-specific differences in OAg expression and fine specificity were found. Those features could not be correlated to clinical presentation, so the same strains may be able to cause both invasive disease and diarrhoea. Whole genome analysis of invasive and gastrointestinal NTS isolates from Africa will provide additional insights regarding whether these strains are the same or different [53].

## Supporting Information

S1 Table a. List of *S*. Typhimurium study isolates and their available basic metadata; b. List of *S*. Enteritidis study isolates and their available basic metadata. (XLSX)

S2 Table a. Serum bactericidal assay (SBA) results for *S*. Typhimurium isolates against pooled Malawian sera. b. Serum bactericidal assay (SBA) results for *S*. Enteritidis isolates against pooled Malawian sera. (XLSX)

S3 Table a. O-acetylation results for *S*. Typhimurium and *S*. Enteritidis isolates. b. Glucosylation results for *S*. Typhimurium and *S*. Enteritidis isolates.
(XLSX)
